# A genome-wide association study of variants associated with acquisition of *Staphylococcus aureus* bacteremia in a healthcare setting

**DOI:** 10.1186/1471-2334-14-83

**Published:** 2014-02-13

**Authors:** Charlotte L Nelson, Kimberly Pelak, Mihai V Podgoreanu, Sun Hee Ahn, William K Scott, Andrew S Allen, Lindsay G Cowell, Thomas H Rude, Yurong Zhang, Amy Tong, Felicia Ruffin, Batu K Sharma-Kuinkel, Vance G Fowler,

**Affiliations:** 1 Duke Clinical Research Institute, Duke University Medical Center, 2400 Pratt Street, Room 0311 Terrace Level, Durham, NC 27705, USA; 2 Center for Human Genome Variation, Duke University School of Medicine, Durham, NC 27708, USA; 3 Department of Anesthesiology, Duke University Medical Center, Durham, NC 27710, USA; 4 Department of Medicine, Duke University Medical Center, Durham, NC 27710, USA; 5 Department of Human Genetics and Institute for Human Genomics, Miller School of Medicine, University of Miami, Miami, FL 33136, USA; 6 Department of Biostatistics and Bioinformatics, Duke University School of Medicine, Durham, NC 27710, USA; 7 UT Southwestern Medical Center, Dallas, TX 75390, USA

**Keywords:** Genomics, Genome-wide association study, Case–control study, Staphylococcus aureus, Bacteremia, Gram-positive bacterial infections, Polymorphism, single-nucleotide, Infections, Nosocomial, Cross infection

## Abstract

**Background:**

Humans vary in their susceptibility to acquiring *Staphylococcus aureus* infection, and research suggests that there is a genetic basis for this variability. Several recent genome-wide association studies (GWAS) have identified variants that may affect susceptibility to infectious diseases, demonstrating the potential value of GWAS in this arena.

**Methods:**

We conducted a GWAS to identify common variants associated with acquisition of *S. aureus* bacteremia (SAB) resulting from healthcare contact. We performed a logistic regression analysis to compare patients with healthcare contact who developed SAB (361 cases) to patients with healthcare contact in the same hospital who did not develop SAB (699 controls), testing 542,410 SNPs and adjusting for age (by decade), sex, and 6 significant principal components from our EIGENSTRAT analysis. Additionally, we evaluated the joint effect of the host and pathogen genomes in association with severity of SAB infection via logistic regression, including an interaction of host SNP with bacterial genotype, and adjusting for age (by decade), sex, the 6 significant principal components, and dialysis status. Bonferroni corrections were applied in both analyses to control for multiple comparisons.

**Results:**

Ours is the first study that has attempted to evaluate the entire human genome for variants potentially involved in the acquisition or severity of SAB. Although this study identified no common variant of large effect size to have genome-wide significance for association with either the risk of acquiring SAB or severity of SAB, the variant (rs2043436) most significantly associated with severity of infection is located in a biologically plausible candidate gene (*CDON*, a member of the immunoglobulin family) and may warrant further study.

**Conclusions:**

The genetic architecture underlying SAB is likely to be complex. Future investigations using larger samples, narrowed phenotypes, and advances in both genotyping and analytical methodologies will be important tools for identifying causative variants for this common and serious cause of healthcare-associated infection.

## Background

Although most persons are colonized with *Staphylococcus aureus* during their lifetimes, only a small percentage will develop infection [1]. The initiation and severity of *S. aureus* infections is complex and influenced by at least 3 characteristics: bacterial virulence factors, host genetic factors, and the environment in which the host and pathogen interact. In previous studies, our group and others have shown the critical role of healthcare contact as the primary environmental risk factor for the acquisition of *S. aureus* infection [2-4]. For example, 85% of patients with invasive methicillin-resistant *S. aureus* (MRSA) infection had healthcare-associated infection [3]. Bacterial genetic characteristics also influence disease type and severity. For example, we have recently shown [5] and externally validated [6] that certain strains of *S. aureus* (e.g., clonal complex 30 and potentially clonal complex 5) are significantly associated with the development of endocarditis and osteoarticular infections.

A variety of research findings suggest that there is a genetic basis for human susceptibility to *S. aureus*. Evidence that human genetic characteristics influence susceptibility to *S. aureus* infection include: 1) higher rates of *S. aureus* infections in distinct ethnic populations, including African Americans [3,7], New Zealand Maori [8], Pacific Islanders [8], Australian Aboriginals [9], and Canadian Aboriginal peoples [10]; 2) familial clusters of *S. aureus* infection [11]; 3) rare genetic conditions associated with susceptibility to *S. aureus*[12-14]; 4) the impact of host genetic variation on persistent *S. aureus* carriage [15]; and 5) variable susceptibility to *S. aureus* infections among inbred mice [16] and cattle [17].

Finally, there is considerable variability within the population of patients who develop *S. aureus* infection, with some patients recovering and others developing a range of complications, including death. Some of this variability can be attributed to the *S. aureus* strain [5], but this does not fully explain the breadth of clinical outcomes observed. Host genetic factors and bacterial genetic factors may interact to influence outcome severity.

While such evidence suggests a genetic basis for host susceptibility to *S. aureus* infection, progress in identifying genes has been slow. Recently, however, several genome-wide association studies (GWAS) have identified variants that may affect susceptibility to infectious diseases such as HIV, viral hepatitis, malaria, tuberculosis, leprosy, meningococcal disease, and Kawasaki’s disease [18], demonstrating the potential value of GWAS in infectious diseases despite some unique challenges (e.g., the role of the pathogen’s genome; the effect of the environment in which the host and pathogen interact).

Thus, the primary goal of the present investigation was to evaluate the association of common genetic variants with acquisition of *S. aureus* infections in humans. To accomplish this, we performed a GWAS on a large cohort of patients with healthcare-associated SAB and a set of controls without SAB but with healthcare contact in the same hospital. A secondary goal of this investigation was to evaluate the effect of potential interaction of the host and *S. aureus* genomes on the severity of clinical outcome. Thus, we performed a secondary GWAS to evaluate the joint effect of the host and pathogen genomes on severity of *S. aureus* infection in the subset of cases for which both clinical records and the *S. aureus* isolate were available.

## Methods

### Study participants

Our study used a case–control design. Data for cases were obtained from the *S. aureus* Bacteremia Group (SABG) repository [2,5], which has prospectively cataloged clinical data, bloodstream *S. aureus* isolates, and human DNA from all consenting patients with SAB within our institution since 1994. Cases (N = 408) were unique adult white inpatients with healthcare-associated SAB [19]. A small number of cases (N = 30) died prior to being consented and thus were included in the repository as anonymous subjects. Although DNA was available for these, no *S. aureus* isolate existed and available clinical data were limited to age, sex, and race. Nonetheless, we felt it was important to include these anonymous subjects in our primary analysis to avoid possible bias induced by exclusion of subjects who died early of their SAB. The anonymous subjects were necessarily excluded from our secondary analysis, which required both the *S. aureus* genotype and clinical data sufficient to define the severity of infection phenotype.

Our study was made both possible and cost-effective due to the availability of previously genotyped data for controls [20], identified through a comprehensive review of electronic health records by an investigator blinded to the genetic data. While potential bias can result from this choice, the available controls were nonetheless appropriate in several ways. First, the controls (N = 779) were unique adult white inpatients undergoing coronary artery bypass grafting (CABG) in the same hospital from which the cases were sampled. Thus, the cases and controls were exposed to the same risk factor (hospitalization) but differed in whether they acquired *S. aureus* infection during their hospital stay. Second, by virtue of undergoing major inpatient surgery, the CABG controls were at increased risk for *S. aureus* infection. The fact that they were at high risk for infection yet remained uninfected makes them suitable controls for this study. Third, while many risk factors are not available for comparison, the comorbid burden in both groups is likely to be considerable. For example, diabetes (a known risk factor for both coronary artery disease and susceptibility to *S. aureus* infection) was common in both populations (37% among the 331 non-anonymous cases; 30% among controls). Further, the known risk factor, age (included as a covariate in our analyses), was similar in both cases and controls (mean 60 years among cases; 59 years among controls). Finally, the genotyping for the controls was performed in the same laboratory using the same methods used for the cases. Assessment of potential bias due to the separate genotyping of cases and controls was performed and is discussed in sections “Genotyping” and “Quality control of genotype data”.

This study was approved by the Duke University Institutional Review Board. All patients provided informed consent according to IRB policies. DNA from the anonymous cases was available from antemortem blood obtained from clinical diagnostic laboratories at the time it was scheduled to be discarded. Use of these samples was fully approved by the Duke IRB under Policies for Decedent Research.

### Genotyping

All genotyping for both cases and controls was performed on the Illumina 610-Quad BeadChip (Illumina, Inc., San Diego, CA, USA) by the Genomic Analysis Facility at Duke University. As described previously, cases and controls were genotyped separately. Thus, to allow detection of potential batch effects, 29 of the original CABG control samples were re-genotyped with the *S. aureus* case samples. These control sample replicates were randomly assigned across all genotyping plates. Additionally, each *S. aureus* plate included 2 case interplate replicate samples and 2 case intraplate replicate samples. All of the expected sample replicate pairs showed greater than 0.99 genotype concordance. One unique copy of each replicated sample was included in the analysis, preferentially retaining the sample with the higher genotyping call rate.

### Quality control of genotype data

DNA samples were excluded for the following reasons: 1) very low intensity or a genotyping call rate <99% as described elsewhere [21], 2) genotypic sex inconsistent with reported sex, 3) unexpected duplicates, and 4) unexpected (‘cryptic’) relatedness. Eleven case samples and 22 control samples failed genotyping and were excluded. The samples included in these analyses had a genotyping call rate between 99.2% and >99.99%. Potential sex mismatches and cryptic relatedness were identified using PLINK software version 1.06 [22], as described elsewhere [21]. Genotypic sex for 5 cases and 3 controls were discordant with reported sex; these individuals were excluded. Samples were evaluated for cryptic relatedness through estimation of identity-by-state (IBS) allele sharing. Four pairs of case samples were shown to be unexpected duplicates. For these pairs, the sample with the lower genotyping call rate was excluded. Additionally, 11 pairs of samples showed excessive cryptic relatedness (IBS allele sharing greater than 0.125), including 2 pairs of 2 *S. aureus* samples, 3 pairs of 1 *S. aureus* sample and 1 CABG sample, and 6 pairs of CABG samples. For the *S. aureus* pairs and the CABG pairs, the sample with the lower genotyping call rate was excluded. For the mixed pairs, the CABG sample was excluded in order to maximize the number of cases for analysis.

SNPs were excluded for the following reasons: 1) low minor allele frequency, 2) excessive missingness, and 3) discordant genotype calls between the original CABG samples and the 29 CABG control samples re-genotyped on the case plates. SNPs with a minor allele frequency below 0.01 (N = 65,160) that were missing in more than 10% of the samples (N = 47,383) or that were discordant in the 29 pairs of CABG control replicates (n = 513) were excluded. Additionally, PLINK was used to confirm that there was no systematic difference in SNP missingness between cases and controls.

### Bacterial genotyping

*Spa* typing and multilocus sequence typing (MLST) were performed on bacterial isolates as previously described [5,23]. PCR oligonucleotide primers for the 7 MLST targets and *spa* have also been described previously [5]. For *spa* typing, eGenomics software (eGenomics, Inc., New York, NY, USA [24]) was used to scan the primary sequence to help identify the orders and names of each repeat. The *spa* type number is representative of the repeat organization. Clonal complexes (CCs) for the isolates were identified via repeat pattern recognition from an existing *spa* type and CC database provided by Drs. Barry Kreiswirth and José Mediavilla, previously confirmed via MLST [23]. Isolates whose *spa* type did not map to a known CC underwent MLST typing. For MLST, the sequence chromatograms for unique alleles were deposited in the MLST database [25]. Alleles at the 7 loci (*arcC, aroE, glpF, gmk, pta, tpi,* and *yqiL*) were used to identify a unique sequence type (ST). MLST allele names and STs were derived from the MLST database [25]. Clonal complexes were assigned to groups of isolates sharing 6 of 7 alleles by using eBURST v3 (Imperial College London) [26,27].

### Population substructure

We assessed population substructure using EIGENSTRAT [28]. Thirty-three control samples and 25 case samples were determined to be population outliers and were excluded from the analysis. After removing these, there were 6 statistically significant (*P-*value <0.05) principal component axes based on Tracy-Widdom thresholds [21,29]. The values for these 6 principal components were included as covariates in our analyses. Our final analysis dataset included 1060 persons (361 cases; 699 controls) with 542,410 SNPs per person.

### Statistical analysis

To identify variants associated with the acquisition of SAB, we performed a logistic regression analysis assuming an additive genetic model, testing the association of the number of minor alleles at each of the 542,410 SNPs with case–control status and correcting for age (by decade), sex, and the 6 significant principal components from the EIGENSTRAT analysis. We compared 361 individuals with healthcare contact who developed SAB to 699 control subjects with healthcare contact but who did not acquire *S. aureus* infection.

To identify variants associated with severity of *S. aureus* infection, we also performed a logistic regression analysis assuming an additive genetic model among the subset of cases for whom complete clinical data and bacterial genotype were available (N = 324). This secondary analysis adjusted for potential effect of bacterial genotype on severity of infection. Persons with SAB due to isolates belonging to either CC5 or CC30 were considered to be at risk for complicated infection, since these bacterial genotypes have been previously associated with endocarditis and osteoarticular infection [5,6]. A joint test (2 degrees of freedom [df]) of host genotype main effect plus interaction with bacterial genotype was performed to test this association [30]. Cases with definite native aortic or mitral valve infective endocarditis (IE), hematogenous bone and joint infections, or both, were defined as having severe (complicated) infections [5]. Patients with complicated infection were excluded if they had a cardiac prosthesis (e.g., pacemaker, cardioverter/defibrillator, prosthetic valve, or mitral valve support ring) or orthopedic arthroplasty. We constructed a binary phenotype for this analysis (cases with severe [complicated] infections versus remaining cases), comparing 76 cases with a severe (complicated) infection to 248 cases who had an uncomplicated infection. Bacterial genotype was also coded as binary (cases with virulent *S. aureus* organisms [CC 5 or 30] versus remaining cases). The full model included the host genotype main effect, the bacterial genotype (CC) main effect, and the host X bacterial genotype interaction. This was compared to a restricted model with only the bacterial genotype main effect. Additionally, age (by decade), sex, dialysis status (1 = patient on dialysis; 0 = patient not on dialysis), and the 6 significant EIGENSTRAT principal components were included as covariates in both the full and restricted models. PLINK software was used for both the primary and secondary analyses.

A Bonferroni correction was applied to adjust the level of significance for multiple comparisons in both analyses. Genome-wide significance was defined as *P*-value <9.2 × 10^-8^ (0.05/542,410). Results were visualized using the WGAViewer tool [31].

### Power

Power calculations were performed for our primary analysis post hoc using the program Genetic Power Calculator [32], assuming an additive model and a disease prevalence of 0.0003 [3].

## Results

### Acquisition of SAB

**Figure 1:**
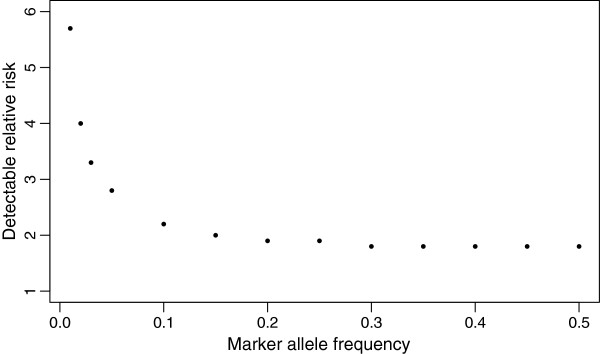
**Power estimates for identification of SAB risk variants.** This graph shows a range of minor allele frequencies and relative risks, assuming a power of 0.8. The calculations were made using Genetic Power Calculator [32], assuming an additive model and a disease prevalence of 0.0003.

After multiple comparisons adjustment of our primary analysis, no SNP met genome-wide significance for association with acquisition of SAB, adjusting for age (by decade), sex, and EIGENSTRAT principal components. This analysis had 0.8 power to detect a genotyped variant with a relative risk (RR) of at least 2.2 and a minor allele frequency (MAF) of 0.1. The minimum RR detectable over a range of possible minor allele frequencies is shown in the Figure 1. Table 1 summarizes the distribution of age and sex for all subjects by case versus control status. All SNPs with a *P*-value <10^-5^ in this analysis are listed in Table 2. The strongest association observed was rs575649 (raw *P-*value = 2.22 × 10^-6^). None of these SNPs were in or near a gene known or suspected to have a role in host susceptibility to infectious disease. Additionally, all SNPs associated with acquisition of SAB in this analysis (*P*-value <10^-4^) were inspected to determine proximity to candidate genes identified through consideration of human orthologs of genes associated with susceptibility to *S. aureus* infection in a murine model [16,33]. None were located in or near a candidate gene of interest identified by the murine analysis.

**Table 1:** Demographic information

	**SAB cases**	**CABG controls**
	**(N = 361)**	**(N = 699)**
Male sex, %	57	76
Mean age at enrollment, y (sd)	60 (14.9)	59 (10.2)

CABG: coronary artery bypass grafting.

SAB: *Staphylococcus aureus* bacteremia.

Data are from patients that were included in the genome-wide association study (GWAS).

**Table 2:** Variants most strongly associated with either acquisition of SAB or severe (complicated) SAB infection, presented in rank order of significance

**SNP**	***P*-value**	**OR**	**L95**	**U95**	**Minor Allele**	**F_A**^**a**^	**F_U**^**a**^	**Chr**	**Coordinate**	**Type**^**b**^	**Closest Locus**^**b**^
Acquisition of SAB											
rs575649	2.22E-06	0.62	0.51	0.76	C	0.398	0.017	6	153215558	Upstream	RP3-468 K3.1
rs1885359	3.18E-06	1.66	1.34	2.05	A	0.342	0.267	22	28011067	Intergenic	RP1-231P7P.1
rs1353492	3.79E-06	2.32	1.62	3.30	A	0.104	0.051	5	92446044	Intergenic	CTD-2091 N23.1
rs13188341	4.10E-06	2.30	1.61	3.28	A	0.104	0.051	5	92443100	Intergenic	CTD-2091 N23.1
rs7068684	5.03E-06	1.82	1.41	2.35	T	0.187	0.123	10	106403115	Intronic	SORCS3
rs7084834	7.53E-06	1.80	1.39	2.32	C	0.186	0.123	10	106403209	Intronic	SORCS3
rs4918120	8.15E-06	1.68	1.34	2.11	T	0.226	0.153	10	106382358	Intergenic	RP11-127O4.2
rs2186567	8.92E-06	0.36	0.23	0.56	C	0.035	0.089	11	70515963	nc_transcript_variant; intron_variant	SHANK2
rs2732986	9.10E-06	1.54	1.28	1.87	C	0.490	0.395	8	5541143	Intergenic	RP11-281H11.1
rs13118964	9.13E-06	0.59	0.46	0.74	T	0.181	0.268	4	161039344	Intergenic	RP11-6C14.1
Severe (complicated) SAB											
rs2043436	1.638E-06	3.85	2.31	6 .41	T	0.283	0.024	11	125925478	Intronic	CDON

F_A: frequency of the minor allele among cases.

F_U: frequency of the minor allele among controls.

L95: lower bound of the 95% confidence interval for the OR.

OR: odds ratio.

SAB: *Staphylococcus aureus* bacteremia.

U95: upper bound of the 95% confidence interval for the OR.

^a^Note that because odds ratios are adjusted for covariates, they cannot be reconstructed directly from the raw allele frequencies reported.

^b^Annotations are from WGAViewer [31].

### Severity of SAB

In our secondary analysis, no SNP met genome-wide significance in a joint test of association with severe (complicated) infection plus interaction with bacterial genotype, after multiple comparisons correction and adjusting for age (by decade), sex, dialysis status, and the 6 EIGENSTRAT principal components. Both the strongest association observed and the only SNP with a *P*-value <10^-5^ in this analysis was rs2043436 (raw *P-*value = 1.64 × 10^-6^), which is included in Table 2. This SNP is located in the *CDON* gene, which encodes a cell surface receptor in the immunoglobulin family. Table 3 summarizes the distribution of bacterial genotype (CC) by complicated versus uncomplicated SAB infection. Again, as performed for the primary analysis of acquisition of SAB, all SNPs associated with severity of SAB in this secondary analysis (*P*-value <10^-4^) were evaluated against the murine model and none were found to be located in or near a candidate gene of interest identified by that analysis.

**Table 3:** Staphylococcus aureus genotype (clonal complex [CC])

**Clonal complex**	**SAB complicated infection**	**SAB uncomplicated infection**
	**(N = 76)**	**(N = 248)**
Virulent *S. aureus* (CC 5 or 30)	50 (66%)	138 (56%)
Unnamed	2 (3%)	4 (2%)
1	3 (4%)	8 (3%)
5	28 (37%)	92 (37%)
8	9 (12%)	43 (17%)
9	1 (1%)	6 (2%)
12	0	6 (2%)
15	4 (5%)	14 (6%)
20	0	1 (<1%)
30	22 (29%)	46 (18%)
45	4 (5%)	17 (7%)
59	3 (4%)	7 (3%)
97	0	2 (1%)
398	0	1 (<1%)
903	0	1 (<1%)

SAB: *Staphylococcus aureus* bacteremia.

Data are from 324 patients included in the secondary analysis of complicated infection. Anonymous cases (N = 30), cases with insufficient clinical data to define severity of infection (N = 4), and cases with missing SA isolate (N = 3) were excluded from this analysis.

To further assess the potential contribution of bacterial genotype on severity of infection, we repeated the secondary analysis, but excluded the bacterial CC and the interaction of SNP with bacterial CC, testing only the impact of host genotype main effect. Thus, this model included the host genotype main effect and the age (by decade), sex, dialysis status, and the 6 significant EIGENSTRAT principal components as covariates. In this analysis, rs2043436 remained the strongest association and was more strongly associated (*P-*value = 2.10 × 10^-7^) with the outcome than it had been when including the interaction with bacterial genotype, suggesting that the host genotype main effect is likely the primary driver of the association of this SNP with severity of SAB infection.

## Discussion

To our knowledge, ours is the first study that has attempted to evaluate the entire human genome for variants potentially involved in the acquisition or severity of SAB. Although this study identified no common variant of large effect size to have a genome-wide significant association with either the risk of acquiring SAB or the severity of *S. aureus* infection, the most highly associated variant (rs2043436, an SNP located within *CDON*) identified in our secondary analysis may provide a biologically plausible target for further study of host susceptibility to severe (complicated) infections. While this SNP is not a strong proxy for a functional variant, there are multiple functional variants in weaker linkage disequilibrium that may be of interest. Given the key role of adhesion to host proteins in staphylococcal pathogenesis, this may be an interesting protein for future studies.

Our inability to identify common variants significantly associated with SAB when there is considerable evidence of a host genetic influence on acquisition of SAB may result from a number of factors. First, our sample size is modest when compared to current GWAS in other diseases. As a result, some signals may not have been detectable at genome-wide significance levels, including those of moderate effect size (RR 1.5–2.2). Thus, our study cannot preclude the possibility that a common variant of moderate to small effect size may be associated with the acquisition of SAB, and such effects may be represented among the many ‘suggestive’ associations (*P-*value <0.00001) detected here. Expansion of the sample set (through meta-analysis or pooled analysis with other cohorts) could resolve this question. Second, given that the clinical spectrum of *S. aureus* bacteremia is quite broad, it is possible that phenotypic heterogeneity may have further complicated our ability to identify variants associated with acquisition of *S. aureus* infection.

Infectious diseases are complex due to interactions among host genetic variants, pathogen genetic variants, and factors in the environment within which the organisms interact. Such interactions present unique challenges for genetic studies. We controlled for the effects of environmental factors by limiting our analysis to cases and controls having the same healthcare exposure. We also controlled for the potential effect of bacterial genetic variability on the severity of infection by including host SNP interactions with virulent *S. aureus* CCs. However, we acknowledge that these approaches may be inadequate to control for mechanisms that are likely much more complex. It is possible that different *S. aureus* genetic variants influence risk for complicated infection and that host variants may interact with these pathogen variants through more complex mechanisms to modify the observed clinical outcomes. It is also possible that non-genetic factors that can influence the likelihood of infection (such as diabetes, corticosteroids, physical fitness, nutrition) could have impacted host susceptibility in our study. Our ability to detect very complex interactions or to adjust for them was limited. Further, our attempt to control for some of these complexities through use of a single-center design engenders a limitation in generalizability as there is potential regional variation in both patient and *S. aureus* characteristics.

It is worth noting that although our control group was matched for environmental exposure, there are potentially important imbalances in clinical risk factors between cases and controls. For example, it is unknown whether there are seasonal or departmental differences in risk of acquisition or severity of *S. aureus* infection. As a result, had genome-wide significant signals been detected, a subsequent analysis to evaluate possible clinical confounding would have been necessary in order to establish the independent association of any finding. Such an analysis, however, would have been complicated both by limited data and clinical exclusions for the control group. Several important risk factors (e.g., active malignancies; dialysis dependency) were exclusions for CABG patients, and therefore could not be included as covariates. Due to the priority to minimize multiple comparisons for our relatively small sample, we felt an adjusted analysis was not warranted in the absence of a genome-wide significant finding.

Finally, while our analysis did not detect a common SNP significantly associated with SAB acquisition, it remains possible that rare genetic variants are modulating susceptibility to SAB. Much of the variation in the human genome occurs at lower frequencies. Indeed, recent reports suggest that rare genetic variants can drive a pronounced clinical phenotype, and are more likely than common variants to have a deleterious effect on a protein coding sequence and potentially cause a disease [34]. Our study had limited ability to detect most rare genetic variation.

## Conclusions

The disparity between exposure, colonization, and infection suggests that humans show varying susceptibilities to acquiring SAB. In this first study to evaluate the entire human genome for variants potentially associated with the acquisition or severity of SAB, no common variant of large effect size achieved genome-wide significance. The genetic architecture underlying SAB is likely to be complex, reflecting oligogenic effects, genetic heterogeneity, and the cumulative effects of multiple rare variants. Future work in which the phenotype is narrowed by focusing on unique subpopulations of SAB (e.g., native valve, endocarditis, or osteoarticular infections) may prove fruitful. Additionally, the use of recent advances in genotyping technology such as Next Generation sequencing is certainly warranted. The cost of whole-genome and whole-exome sequencing is falling rapidly, and such methods provide the advantage of near-complete coding sequence variant identification. It would be especially interesting to sequence those persons who have a more “extreme” clinical outcome, since it might be easier to identify causative variants in such a population. Investigations using larger samples and advances in both genotyping and analytical methodologies will be crucial to success in unraveling the causal pathway for this common and serious cause of healthcare-associated infection.

## Abbreviations

CABG: Coronary artery bypass grafting; CC: Clonal complex; df: Degree of freedom; GWAS: Genome-wide association studies; IBS: Identity-by-state; IE: Infective endocarditis; MAF: Minor allele frequency; MLST: Multilocus sequence typing; MRSA: Methicillin-resistant *Staphylococcus aureus*; SAB: *Staphylococcus aureus* bacteremia; SABG: Staphylococcus aureus Bacteremia Group; SNP: Single-nucleotide polymorphism; ST: Sequence type.

## Competing interests

VGF served as Chair of V710 Scientific Advisory Committee (Merck); has received grant support from Merck, Cerexa, Pfizer, Novartis, Advanced Liquid Logics, MedImmune, and the National Institutes of Health; has been a paid consultant for Merck, Astellas, Cubist, Cerexa, Durata, Pfizer, NovaDigm, Novartis, Medicines Company, Biosynexus, MedImmune, Galderma, and Inimex; and has received honoraria from Merck, Astellas, Cubist, Pfizer, Theravance, and Novartis. No other authors have any commercial or other association that might pose a conflict of interest.

## Authors’ contributions

VGF conceived of the study idea, participated in the design, secured the grant funding, oversaw the execution of the study, and drafted the manuscript. CLN conducted the primary analysis and drafted the manuscript. KP participated in the primary analysis and drafted the manuscript. MVP provided the genotypic data for the control patients. WKS and ASA oversaw the analytical aspects of the study. SHA performed genetic analyses in the murine model. THR performed amplification of DNA and bacterial genotyping. AT, YZ, and LGC prepared genomic samples for analysis. FR was responsible for enrollment of subjects and data collection. BKS-K prepared genomic samples for analysis and participated in genetic analyses in the murine model. All authors read, edited, and approved the final manuscript.

## Pre-publication history

The pre-publication history for this paper can be accessed here:

http://www.biomedcentral.com/1471-2334/14/83/prepub
